# Sleeplessness

**Published:** 1875-02

**Authors:** 


### SLEEPLESSNESS.

A symptom of mental exhaustion, indicative of a very great degree of mental strain, is persistent wakefulness. The physiological cause of this condition is well understood. During excessive labor of the brain there is an increased flow of blood to the working organ, the vessels of the head and neck become distended with blood, as is shown by the flushing of the face. If this condition of distention is long continued, the vessels are apt to lose the power of contracting when mental activity is diminished. Hence arises the impossibility of fulfilling the physical conditions of sleep, the most important of which is the diminution of the flow of the vital fluid to the brain. Some extraordinary instances have been recorded of prolonged wakefulness as a result of mental overstrain. Boerhave mentions that when on one occasion intently engaged on a particular study, he did not close his eyes in sleep for six weeks. Sir Gilbert Blane was informed by General Pichegrue that for a whole year, when engaged in active campaign, he slept but one hour in the twenty-four. These and other similar cases, have probably been unconsciously exaggerated, for people often sleep without having an after-consciousness of the fact. It is certain that the continued deprivation of any considerable part of the normal amount of sleep will be seriously detrimental to health. There is the case of a literary man in America who, for nearly a year, while intently engaged in a favorite study, restricted his period of rest to four hours a day, and frequently less. At the end of that time the over-tasking of his mental powers was manifested in a curious way. He told the physician that, though still able to maintain a connected line of reasoning, he found that as soon as he attempted to record his ideas on paper the composition turned out to be simply a tissue of arrant nonsense. When in the act of writing, his thoughts flowed so rapidly that he was not conscious of the disconnected nature of what he was writing; but as soon as he stopped to read it over he was aware how completely he had misrepresented his conceptions. If the language happened to be at all intelligible, it was sure to have no relation to the ideas he wished to express. Thus, wishing to obtain a book from a friend, he found that, instead of the request, he had written the prayer of Socrates, as given by Plato.

Sir Isaac Newton, in the later years of his life, suffered greatly from wakefulness. The fact, well known to every medical man, that persistent sleeplessness is frequently the precursor or initiatory stage of several most intractable maladies, physical and mental, always invests the presence of this indication of mental overstrain with grave interest. But a continued course of excessive mental labor generally manifests itself on the mind itself in various ways, all more or less premonitory of approaching collapse. The brain-worker begins to perceive an unwonted want of clearness in his ideas; work becomes gradually irksome; he is alarmed at sudden awkward failures of memory; a feeling of surfeit or disgust will steal over him in the midst of work; he becomes unable to fix his attention, and latterly feels as if all mental energy was crushed out of him.

If these warnings of an overwrought brain are disregarded, the wonder frequently is, not that the inevitable retribution follows, but that it should have been so long delayed. What particular form the Nemesis shall assume, whether of physical or mental disease, will be determined by accidents partly of personal habit and temperament, and partly of inherited predisposition. It is noteworthy, however, that the common opinion that excessive mental occupation gravitates toward insanity, does not appear to be verified by facts. Indeed, one of the foremost of living physicians doubts whether alienation of mind is ever the result of over-strain. It is to physical, not to mental derangement, that excessive work of the brain generally gives rise. Insanity, as we have before remarked, finds the most suitable material for its development among the least educated classes; while the worst form of physical diseases are originated and intensified by our educated, over-straining brain-workers.

The cure for this sleepless condition is simple, although the treatment must be radical. The first essential is to abandon the particular class of brain exercise which has induced or attended upon the difficulty. The next thing is give up coffee, tea, tobacco, and all stimulants. To use any form of alcohol in such a case may be suicide. Finally, live out of doors, exercise daily till you are tired out, and then go to bed in a quiet, well-aired, cool room. In a month you will be well, and may go on in the old brain-wearing, destructive way, if you have not learned the lesson of prudence by suffering.

